# Overtreatment of malaria in the Nigerian healthcare setting: prescription practice, rationale and consequences

**DOI:** 10.11604/pamj.2023.45.111.31780

**Published:** 2023-06-29

**Authors:** Emmanuel Temitope Anjorin, Olufemi Nicholas Olulaja, Moyosoore Emmanuel Osoba, Oluwafemi Temitayo Oyadiran, Ayodele Oloruntoba Ogunsanya, Omotola Nofisat Akinade, Jemimah Mayowa Inuojo

**Affiliations:** 1 Lagos State University Teaching Hospital (LASUTH), Ikeja, Lagos, Nigeria,; 2 African Center of Excellence for Genomics of Infectious Diseases (ACEGID), Redeemer´s University, Ede, Osun State, Nigeria,; 3 Medical Research Council Unit, London School of Hygiene and Tropical Medicine, Fajara, The Gambia,; 4 Infectious Disease Hospital (Mainland Hospital), Yaba, Lagos State, Nigeria,; 5 Ikorodu General Hospital, Ikorodu, Lagos, Nigeria,; 6 Xcene Research (Contract Research Organization (CRO)), Ikeja, Lagos, Nigeria

**Keywords:** Malaria, overtreatment, consequences

## Abstract

Nigeria is one of the countries in the world with the highest burden of malaria, accounting for a quarter of all cases in Africa. According to the Centers for Disease Control and Prevention, microscopic examination remains the gold standard for laboratory confirmation of malaria. However, the policy and practice of presumptive treatment of malaria for all febrile illnesses has been widely advocated in sub-Saharan Africa. Presumptive management of fevers and/or other symptoms of malaria results in over-diagnosis, and consequently overtreatment. This article discusses the overtreatment of malaria as practiced in Nigeria and other African regions against standard treatment guidelines, highlights a wide range of its associated effects on patients and proffers possible solutions to curb the unethical practice of malaria overtreatment.

## Essay

Malaria is an infectious disease caused by the plasmodium parasite and is spread through the bite of its vector- an infected female Anopheles mosquito. Malaria, being a disease of global health concern, was estimated to affect 229 million people with over 400 thousand deaths worldwide in 2019 [1]. This febrile illness is generally referred to as a disease of poverty. Globally, the incidence of malaria is concentrated in the world´s poorest nations with 90% of malaria mortality occurring in sub-Saharan Africa [2]. Nigeria is one of the countries in the world with the highest burden of malaria, accounting for a quarter of all cases in Africa [3]. It is estimated that more than half of Nigeria's population experiences at least one episode of malaria each year, accounting for approximately 30% of outpatient visits, 20% of all hospital admission, and 10% of hospital deaths [3]. In Nigeria, malaria contributes to the economic burden of people living in communities where it is endemic. According to the World Health Organization, microscopic examination remains the gold standard for laboratory confirmation of malaria [4]. However, the policy and practice of presumptive treatment of malaria for all febrile illnesses has been widely advocated in sub-Saharan Africa. Presumptive management of fevers and/or other symptoms of malaria results in over-diagnosis and consequently overtreatment [5]. While the rationale for malaria overtreatment in the Nigerian healthcare largely hinges on the good intentions of medical practitioners, overtreatment of malaria may have dire consequences on the end users. In light of the decline in the incidence and mortality rate of malaria in recent years [5], it is therefore imperative to curb the practice of over-diagnosis and overtreatment of malaria in endemic countries such as Nigeria to prevent the burgeoning of another public health menace.

**Burden:** the public health challenge as well as the socio-economic burden of malaria is enormous especially in African countries. Under-five children are the most defenseless group affected by malaria, as they accounted for 61% of the 266 000 malaria deaths worldwide in 2017 [6]. To further demonstrate the enormity of the situation, an estimated US$3.1 billion was invested in malaria control and eradication globally in 2017, by governments of malaria endemic countries and international partners; an amount slightly higher than the figure reported for 2016. Nearly three-quarters (US$ 2.2 billion) of this investment in 2017 was spent in the African Region [7]. One of the most common causes of death in Nigeria is malaria and every Nigerian falls ill of malaria at least three times per year [8]. Malaria remains the number one reason people fall sick and die in Nigeria, accounting for one-fifth of mortalities [9]. The emergence of several resistant strains of mosquitoes has made diverse efforts at reducing the burden of the infectious disease futile. While African countries like Morocco and Algeria have successfully controlled and rid their environment of malaria through adherence to guidelines by international agencies like the World Health Organization, Nigeria has experienced a spike in the endemicity of malaria over the past few decades, contributing the heaviest burden of malaria in 2020; one out of every four cases and 19% of deaths globally [10]. In 2017, the world malaria report states that Nigeria accounted for 27% of the 445,000 global deaths from malaria [9]. The situation worsened five years later according to the 2022 world malaria reports, as over 50% of deaths globally due to malaria occurred in four major countries; of which Nigeria sits atop with 31.3%. Additionally, Nigeria recorded 38.4% of malaria deaths amongst children under five years worldwide [11]. Usually, pregnant women are largely predisposed to severe anemia, especially in specified areas where malaria prevalence is very high in Nigeria. Occurring more amongst primigravidae, these groups are highly susceptible to malaria due to their low immunity. Whilst 300,000 people died of conditions related to malaria in Nigeria in the year 2010, 11% of maternal deaths were directly as a result of malaria in pregnancy [12].

**Presentation and diagnosis:** malaria is an acute febrile illness with an incubation period of 10-14 days but varies to over 30 days. Children are more prone to severe forms of illness and have worse symptoms than adults. Malaria typically presents in paroxysm which usually begin with a bout of headache, fever and chills alternating with symptoms-free period with each attack lasting 4-6 hours every 48-72 hours. Clinical symptoms of uncomplicated malaria include fever, chills, sweats, headaches, nausea, vomiting, and body aches. Other symptoms occasionally seen are diarrhea, abdominal pain, and cough [13]. Diagnosis based on clinical features alone has been shown to have low specificity therefore establishing diagnosis should be based on laboratory confirmation [14]. Light microscopy is the investigation of choice with a high specificity and sensitivity rate, and it involves the observation of plasmodium species in Giemsa-stained slides [13]. However, in resource poor centers and out-of-hospital centers, rapid diagnostics test kits (RDT) have been shown to have high specificity if used appropriately [15]. According to the World Health Organization (WHO), there should be prompt parasitological confirmation either by microscopy or rapid diagnostic test (RDT) for all patients with suspected malaria prior to commencement of treatment [4]. However, treatment may be based on the clinical suspicion of malaria when there is non-availability of RDT or microscopy. Microscopy with visualization of Giemsa-stained parasites is the gold standard for the diagnosis of malaria [4].

**Standard treatment guidelines:** for patients (children and adults) presenting with uncomplicated malaria, the World Health Organization (WHO) recommends treating with a three-day course of artemisinin-based combination therapy (ACT) except pregnant women in their first trimester who should have seven days of quinine and clindamycin [4]. The available artemisinin-based combination therapies are artemether + lumefantrine, artesunate + amodiaquine, artesunate + mefloquine, dihydroartemisinin + piperaquine, and artesunate + sulfadoxine-pyrimethamine (SP). Second-line treatment includes alternative ACT known to be effective in the region; or seven days course of artesunate plus tetracycline or doxycycline or clindamycin; or seven days course of quinine plus tetracycline or doxycycline or clindamycin [4]. Parenteral artesunate is the recommended first-line drug for treatment of severe malaria. The patient is administered artesunate for at least 24 hours until they can tolerate oral therapy, then a three-day course of oral ACT is instituted. In a resource-limited setting where artesunate is not available, artemether is preferred to quinine for the treatment of severe malaria [4].

**Misdiagnosis and mismanagement of malaria in Nigeria:** in Nigeria, malaria diagnosis and management have been trivialized as many non-clinicians assume any symptom of fever to be malaria. A study conducted in southeastern Nigeria found that 86% of caregivers diagnosed malaria mainly by noticing fever, headache, cough, and pains [16]. A study in Sokoto State examined 800 febrile children who were presumed to have malaria and found that only 56% had confirmed malaria while the remaining 44% were misdiagnosed and treated for malaria [17]. In Borno State, 310 febrile patients were tested and only 1% were positive for malaria. While 48% tested positive for malaria and one other arbovirus infection, while the remaining had infections other than malaria [18]. Although malaria is prevalent in Nigeria, the misdiagnosis of other febrile illnesses as malaria erroneously contributes to its burden. In another part of the country, a study evaluated 2,171 records of patients managed for uncomplicated malaria and found that only 480 had laboratory confirmation of malaria and the rest were given presumptive diagnosis of malaria and subsequently treated, with no laboratory evidence [19]. More recently, hypervitaminosis A was misdiagnosed as malaria in a 7-year-old boy who presented with vomiting, headache, generalized body weakness, and blurred vision [20]. Despite WHO´s guideline requiring laboratory confirmation of malaria diagnosis prior to treatment, there are overwhelming cases of presumptive diagnosis by clinicians, caregivers, and patent medicine dealers. Mismanagement of malaria cases expectedly follows many cases of its misdiagnosis [19]. It is spectacularly common among individuals who self-medicate, and those who patronize patent drug dealers. Self-management of malaria is common, accounting for as high as 51% of malaria management and many adults self-medicate on antimalarial twice or more times in a year [21]. A study conducted in Enugu, Southeastern Nigeria, examined 1,012 cases of malaria and reported that 98% received antimalarial drug prescription with no prior review of malaria tests [22]. Co-prescription of antibiotics is common during management of malaria and may be as high as 50% of cases of presumptive malaria [19]. Nearly 92% of those who sought malaria treatment made their own diagnosis or had their friend or family member make the diagnosis for them [3]. Overall, mismanagement is commoner among adults compared to children because caregivers of sick children are more likely to receive doctor´s prescription prior to treating their wards with antimalarial medication [23].

**Overtreatment of malaria:** malaria diagnosis and treatment are critical, as malaria symptoms can worsen and cause serious complications including death. However, as the cases began to decrease in the tropics, the WHO now recommends that all suspected cases must be confirmed by microscopy or alternatively by rapid diagnostic testing. Despite this guideline, fever is still largely attributed to malaria and treated empirically with medications [24] giving rise to unnecessary administration of antimalarial drugs to individuals who do not have it. A study conducted in a primary health center in Lagos, Southwest Nigeria revealed an overtreatment percentage of 83.1% among under 5s [5]. Similarly, a study conducted in a tertiary health facility in Zamfara, Northern Nigeria revealed that 79% of study participants had received antimalarial medications without testing prior to presentation at the facility [25]. This problem is not peculiar to Nigeria as a study conducted in a Ghanaian healthcare center revealed that 84.1% of malaria negative cases and 78.2% of children with no malaria test were given antimalarial medications [1]. Similarly, a study conducted in Homa Bay, Kenya revealed that 57.2% of persons with negative malaria test were given antimalarials [24]. Malaria overtreatment can result from treating symptoms without performing tests, and administering treatment for negative test results. Additionally, there is a possibility that doctors in the private sector overtreat malaria in order to raise revenue and meet up with the financial demands of the facility. However, research has also shown that overtreatment of malaria can also be due to patients who mount pressure on doctors to prescribe antimalarials against the physician´s better judgement [26].

**Consequences of overtreatment:** consequent to the problem of overtreatment, there are corresponding overwhelming implications which pose serious health risks and economic relevance. Failure of the Nigerian healthcare systems to prioritize parasitological confirmation provides major evidence of enormous overdiagnosis and overtreatment with antimalarial combination therapies over the years [5]. By normal progression, overdiagnosis of malaria is a poor diagnosis and would naturally be followed by a wrong choice of treatment for the patient´s illness, which prevents the prompt delivery of accurate and efficacious management of the patient´s condition [5]. Malaria stands as an intricate health condition especially among children, but its frequent overdiagnosis and consequent overtreatment results in negligence of other possible fever-associated life-threatening conditions. This connotes those other causes of febrile illness, such as pneumonia and meningitis, are missed ab initio at health centers until weeks or months later when no notable improvement is seen in the child's health [27]. In regions of high malaria endemicity, physicians presumptively manage ill children due to their distrust for negative outcomes of malaria parasite tests, or because of the fear that they might miss potential cases of severe malaria with imminent deadly complications [28]. Consequentially, febrile patients with severe symptoms and signs who live in communities with high malaria prevalence are continually being treated using antimalarial medications, despite negative test results. This counters the current test-treat policy of the WHO and might culminate in failure to accurately diagnose and provide treatment for other unconventional causes of fever, other than malaria [28]. Hence, overtreatment of malaria indirectly contributes to rising rates of morbidity and mortality mostly among the vulnerable members of the society because less attention is given to other fever-related illnesses, asides malaria [27]. Other than failure to give early treatment for other life-threatening diseases, the treatment of all malaria-related complaints as malaria, based on clinical suspicion accounts for wastage of valuable resources especially among people of low socio-economic power [27]. In Malawi, very poor households spent over a third of their annual family income on treatment of malaria either directly or indirectly compared to 4.2% of annual income spent by the elites who have higher earning power. Unfortunately, some households even spent over 10% of their yearly family revenue per malaria treatment episode in Sri Lanka [18]. So, it is obvious that people of the lowest economy in communities pay remarkably more for these healthcare services, just to ensure that accurate and quality treatment is administered. As a result, there would be an increased number of visits to hospitals and clinics, with a resultant rise in cumulative cost of care for patients. Hence, prioritization of accurate malaria diagnosis and appropriate treatment at the primary level of healthcare in Nigeria remains crucial to the improvement of health outcomes and possibly downturn of poverty [29]. Additionally, the high cost of treatment of malaria remains glaring but seems to have been tolerated in the past due to availability of cheap first-line medications like chloroquine. However, the introduction of ACTs which are more expensive antimalarial medications calls for a clear focus on the consequences of malaria misdiagnosis, in order that malaria drugs are given only to persons who have the illness and to ensure that the financially challenged citizen's benefit more from this effective medicine [29]. Artemisinin-based combination therapy are costlier than the previous first-line medications such as sulphadoxine-pyrimethamine and chloroquine and prescribing them for ailments that are not truly malaria would be an obvious waste of funds and create a terrible effect on health outcomes [29]. Drug resistance stands out amongst the consequences of malaria overtreatment. This is because injudicious prescription of ACTs would cause gross wastage of this medication and potentially increase the possibility of developing resistance. More so, treating malaria blindly without confirmation of plasmodium parasitemia contradicts standard practices which makes patients prone to antimalarial resistance [5]. Resistance to antimalarial medication can also worsen because patients take antimalarial drugs for other infectious conditions presenting with the same symptoms as malaria. Consequently, patients use antimalarial drugs unnecessarily, raising the chance of resistance. On the other hand, drug resistance creates an avoidable vicious cycle of patients returning to medical facilities occasionally, for the same complaints [29].

**Recommendations to improve appropriate diagnosis and management of malaria:** amidst the several effects of overtreatment, possible interventions abound. Ensuring quality malaria rapid diagnostic tests (RDTs) increases the chance of effective and timely malaria case management in the long run. Hence, rehabilitation of the country´s diagnostic system, ensuring availability of RDTs, training of health workers, infrastructural improvement, and quality assurance for diagnostic processes, etc., are important for effective diagnosis of malaria [5]. The application of the WHO´s universal directive on parasitological confirmation of malaria has been adapted by most countries in Africa and provides a platform for the improvement of health care delivery by inculcating scientifically proven practices. Hence, health workers should be given sufficient quality tools for diagnosis to improve confirmation of malaria parasitemia before treatment [5]. Another way to curb overtreatment would be fostering initiatives that strengthen health systems and ensuring standard management guidelines are given utmost priority, especially in areas where malaria is highly prevalent [24]. Effective training for health workers and health education for patients could possibly change their attitude towards the diagnosis and management of malaria. Organizing concise trainings for medical teams on the use of RDTs and a well detailed training for patients at community level would curb misdiagnosis and overtreatment of malaria (Table 1).

**Table 1:** summary of recommendations to improve diagnosis and management of malaria

S/No	Recommendations to improve appropriate diagnosis and management of malaria
1	Availability of rapid diagnostics test kits and training of health workers on how to effectively use them
2	Quality sufficient equipment should be provided to health workers for confirmation of malaria parasitemia before treatment
3	Creating and supporting initiatives that strengthen health systems and ensure standard management guidelines
4	Health education for patients and their relatives could affect their attitude towards the diagnosis of malaria and use of antimalaria medications
5	Training for medical teams on diagnosis of malaria and quality assurance; which ultimately impact the patients and the communities they serve

## Conclusion

To conclude, the universal policy for malaria diagnosis is an evolving process that needs time and substantial resources to attain absolute implementation across the sub-Saharan region of Africa. For this process to succeed, contributions would be required from all sectors, either private or public. While universal standard guidelines exist, the Nigerian healthcare system must create a workable plan for compliance to the treatment guidelines for malaria, which would in turn help to curb the problem of overtreatment. This plan must involve both the private and public stakeholders of the country’s health system to achieve the needed impact.
